# Antifungal Styryloquinolines as *Candida albicans* Efflux Pump Inhibitors: Styryloquinolines are ABC Transporter Inhibitors

**DOI:** 10.3390/molecules25020345

**Published:** 2020-01-15

**Authors:** Wioleta Cieslik, Joanna Szczepaniak, Anna Krasowska, Robert Musiol

**Affiliations:** 1 Institute of Chemistry, University of Silesia, 75. Pułku Piechoty 1, 41-500 Chorzów, Poland; wioleta.cieslik@us.edu.pl; 2 Department of Biotransformation, Faculty of Biotechnology, University of Wroclaw, Joliot-Curie 14a, 50-383 Wroclaw, Poland; joanna.szczepaniak@uwr.edu.pl (J.S.); anna.krasowska@uwr.edu.pl (A.K.)

**Keywords:** styrylquinolines, *Candida albicans*, ABC transporters, Cdr1p

## Abstract

Styrylquinolines are heterocyclic compounds that are known for their antifungal and antimicrobial activity. Metal complexation through hydroxyl groups has been claimed to be a plausible mechanism of action for these types of compounds. A series of novel structures with protected hydroxyl groups have been designed and synthesized to verify the literature data. Their antifungal activity against wild-type *Candida albicans* strain and mutants with silenced efflux pumps activity has been determined. Combinations with fluconazole revealed synergistic interactions that were dependent on the substitution pattern. These results open a new route for designing active antifungal agents on a styrylquinoline scaffold.

## 1. Introduction

Invasive mycoses are an important public health problem, especially for immunosuppressed patient. *Candida albicans* is the one that is most often associated with serious invasive fungal infections, and it is responsible for 8–10% of the cases with a high mortality rate (30–80% depending on the patient) [1]. Treatment of fungal infections is markedly limited by the problems of drug safety and fungal resistance. Among the drugs used in antifungal treatment, echinocandin is used for invasive candidiasis while voriconazole and isavuconazole are the drugs of choice for aspergillosis. Other drugs from the conazoles group, like fluconazole (FLC), have rather limited use due to the fact of resistance. Another drug with a wide spectrum of activity is amphotericin B (AmB). Amphotericin B belongs to a group of polyene antibiotics that act by forming an oligomeric pore structure within the fungal plasma membrane by interacting with the membrane sterols. This leads to a flux of cations, membrane depolarization, and cell death [2]. Amphotericin B can also bind to cholesterol in mammalian cells and can cause severe cellular toxicity [3,4]. The mode of action of the azole derivatives is based on inhibiting the ergosterol biosynthesis pathway at different stages [5]. Ergosterol is the major component of the fungal cell membrane; hence, it is one of the targets for antifungal drugs [6]. However, the growing resistance of fungi to azoles is a problem. *Candida albicans* has developed many drug resistance mechanisms. One example is the overexpression of the drug transporters (e.g., ATP binding cassette (ABC) pumps—*Candida* drug resistance (Cdr1p and Cdr2p). This mechanism is not very specific; ABC transporters have the ability to export many structurally dissimilar compounds, for instance, azoles, among others [7].

**Figure 1:**
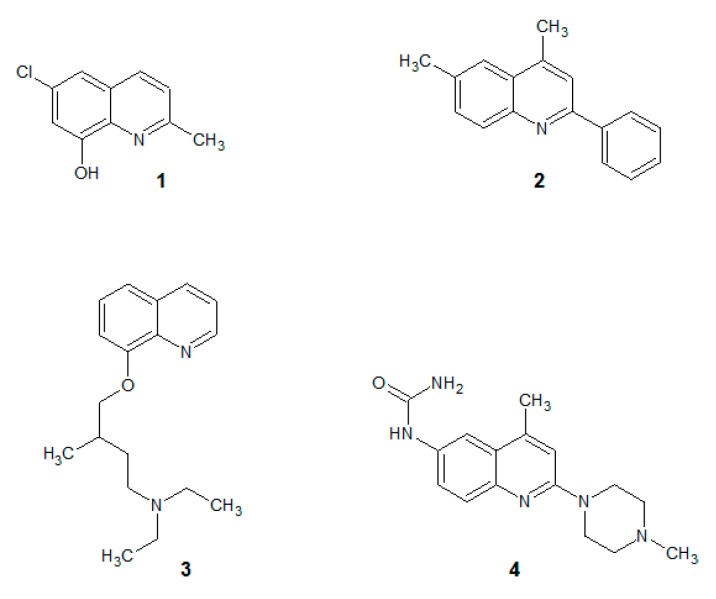
Antifungal quinolines. (**1**): oxine derivative; (**2**): 2-phenylquinoline; (**3**): alkylated oxine; (**4**): 2-morpholine derivative.

Our team has been investigating the quinoline derivatives for their biological activity for several years. For example, anticancer [8,9], antibacterial [10,11] and antifungal [12,13,14] quinolines have been described during our research. In general, the idea of small-molecule quinolines that have an antifungal activity has been known from the works of Gershon [15,16,17,18]. At the beginning, compounds that were described were based on small quinolines that were substituted with halogens and preferably with the 8-hydroxy group (Figure 1(**1**)). Their activity was non-specific and covered a broad range of fungal strains. These initial examples were later developed into more sophisticated molecules that had a more specific mechanism of action.

For example, several quinolines were found to be inhibitors of the fungal cell wall synthesis (Figure 1(**2**)) [19]. They are lytic for microbial cells (Figure 1(**3**)) [20] including the *Candida* strains [21,22] and cause an accumulation of endogenous reactive oxygen species in *C. albicans* biofilms (Figure 1(**4**)) [23].

**Figure 2:**
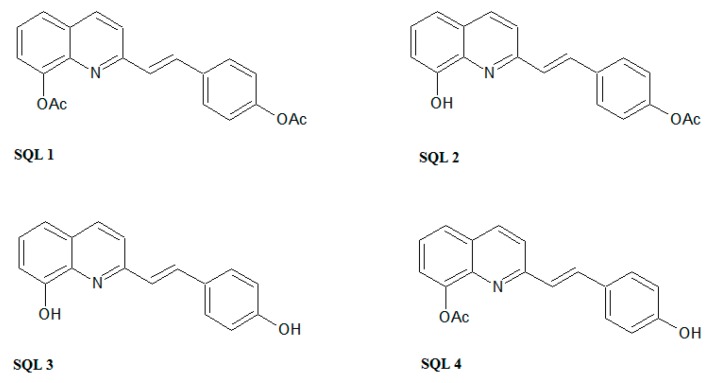
The styrylquinolines (SQLs) that were synthesized in this study.

In this study, we present styrylquinolines (SQLs) that have promising, novel antifungal properties. Styrylquinolines are interesting lipophilic compounds that were initially obtained as analogues of allylamines [19], although their spectrum of activity is wider, covering also anticancer [24], antibacterial [10], and antiviral activity [25,26]. Interestingly, favorable patterns of substitution vary among these activities. The 8-hydroxyquinoline core prevails in most of those applications but other positions are more specific and vary among types of activity. For example, for antiviral activity, SQLs should have electron-donating groups in the styryl part of the molecule and particularly effective are 3,4-dihydroxyl or alkoxyl groups [25,27]. The contrary is beneficial for anticancer activity where resonance positions (2-, 4-) in the styryl moiety should be occupied by electron-withdrawing groups such as cyano or nitro [9]. In antifungal activity, particularly high activities may be achieved by additional electron-accepting groups in quinoline moiety such as in 5,7-dichloro-8-hydroxyquinoline [10]. This may be associated with ability to chelate metal ions, that was primarily established as the mechanism of action of small molecule quinoline antifungal agents [28,29]. Otherwise, the structure–antifungal activity relationship for substitution in styryl rings is not yet fully explored. According to our previous works as well as the literature data, the halogen and hydroxyl (but not alkoxyl) substituents at position C4 seems to be effective [10,30]. Therefore, we decided to investigate whether the free hydroxyl group is essential for this activity (Figure 2).

## 2. Results

**Figure 3:**
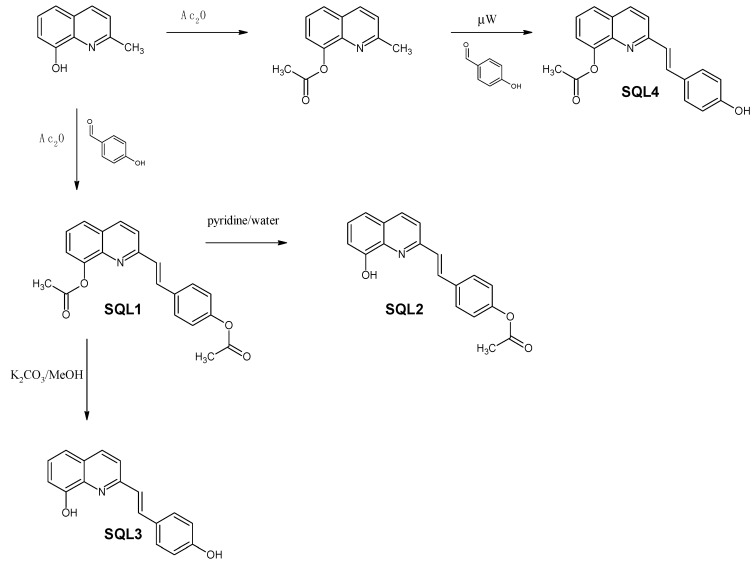
Synthesis of the styrylquinolines.

In order to produce all of the combinations of acetyl/hydroxyl derivatives, we used selective hydrolysis and the modified methods that are typically used for styrylquinoline synthesis as is depicted in Figure 3.

Commercially available 8-hydroxy-2-methylquinoline was reacted with acetic anhydride to acetylate the hydroxyl group. The resulting 8-acetoxyquinaldine was converted into **SQL 4** under microwave irradiation according to the modified method that was reported for other SQLs [31]. Similarly, 8-hydroxy-2-methylquinoline combined with 4-hydroxybenzaldehyde was refluxed in an acetic anhydride/acetic acid mixture for 18 h to produce **SQL 1**. Both hydroxyl groups were acetylated in this reaction environment. Then, the acetic anhydride was evaporated under a vacuum. A pyridine/water mixture was added to the residue and refluxed for 3 h, which resulted in selectively deacetylated **SQL 2**, while the hydrolysis of **SQL 1** in methanolic K_2_CO_3_ produced **SQL 3**.

### 2.1. Antifungal Activity

To assess the antifungal activity of the styrylquinolines that were investigated, we determined the minimal inhibitory concentration (MIC) values for the wild-type *C. albicans* strain. Furthermore, we selected single and double *cdrΔ* mutants (Table 1) in order to gain insight into the chance of resistance.

Compounds **SQL 4**, **SQL 1**, and **SQL 2**, which did not have or had one hydroxyl group, had a rather weak antifungal activity. It is worth noting that the position of the OH/AcO group seemed to be irrelevant as both of the SQLs that had only one OH group that was unprotected had the same MIC level as **SQL 1** (Table 2). The **SQL 3** had a strong antifungal effect, especially on the *cdr1Δ* strain. Control compounds 8-hydroxyquinoline and 8-hydroxyquinaldine had a similar or worse effect on the strains (Table 2).

The simultaneous applications of two or more compounds is a good strategy against resistant strains. The synergistic action of the mixtures of drugs can overcome resistance even with apparently inactive substances [32]. To measure interactions of the SQLs with FLC, we determined the fractional inhibitory concentration (FIC) for the drugs that were tested separately and in combinations. Next, we estimated the FIC index for all of the interactions. These indexes can be used to determine interaction types among two or more drugs that are used simultaneously. In general, FICs below 0.5 can be considered as synergistic as presented in Section 4.3. We found a synergism between **SQL 3** and FLC against *C. albicans* (Table 2) but no difference in the FIC between the wild-type strain and the mutants which indicates a non-specific activity of **SQL 3** and FLC to the ABC pumps (Table 2). We found a four-fold weaker FIC interaction for **SQL 2** for the *cdr1Δ* and *cdr1Δcdr2Δ* strains than for the rest of strains. This compound as well as FLC separately were ejected from the *Candida* cell by the ABC transporters, but when the cells were treated with a combination of drugs, their export decreased due to the competitive inhibition of Cdr2p, hence the low FIC for the *cdr1Δ* and *cdr1Δcdr2Δ* mutant. A similar situation was observed in the case of **SQL 4** for which the synergy was only apparent in the strains that had one or both of the transporters deleted—when both of the Cdr pumps were expressed, they complemented each other’s activity and no synergistic action was observed.

### 2.2. The Rhodamine 6G Efflux Assay

**Figure 4:**
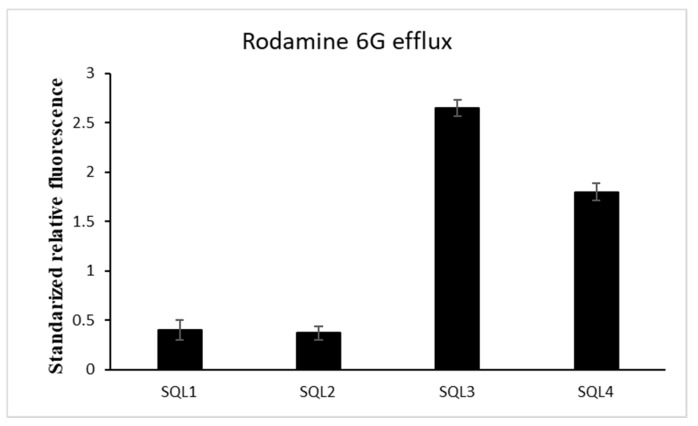
Styrylquinolines (½MIC) influence on energy-dependent rhodamine 6G efflux 30 min after efflux was induced by adding glucose. (±SD, *n* = 3).

In order to determine whether SQLs influence ABC transporters in *C. albicans*, we investigated the activity of the efflux pumps using an R6G assay. All of the compounds were applied at a concentration that was below their antifungal activity concentration to maintain living cells during the experiment. The **SQL 4** had a weak influence on the R6G leakage from the cells (Figure 4). Despite the high synergism of **SQL 3** with FLC, we did not observe any inhibition of the pumps removing the R6G from the cells (Figure 4). We also observed differences between the synergisms of **SQL 1** and **SQL 2** with FLC and their influence on the R6G efflux from the cells (Table 2, Figure 4).

### 2.3. The Influence of Styrylquinolines on the Cdr1p Level

**Figure 5:**
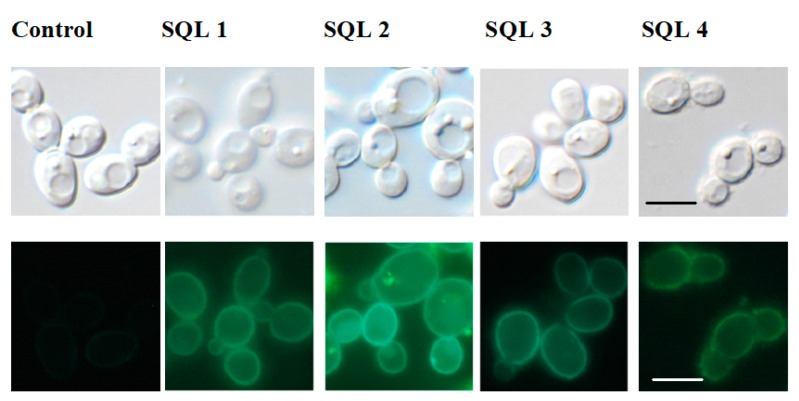
Microscopic imagining of the *Candida albicans* cells. Top panels: differential interference contrast; bottom panels: Cdr1p-GFP. Scale bars = 10 μm.

**Figure 6:**
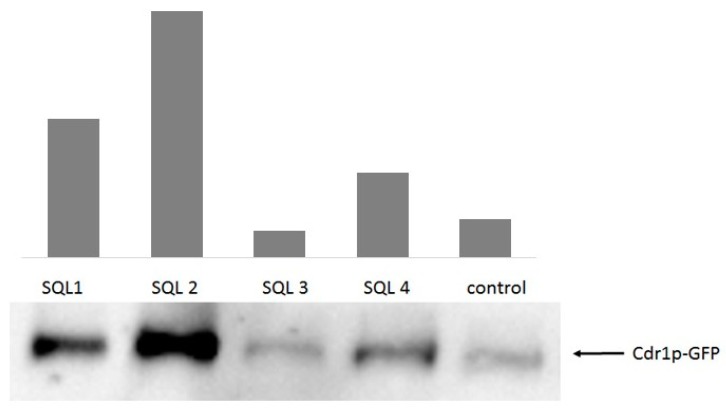
Cdr1p-GFP protein levels in crude protein extracts of the cells after treatment with styrylquinolines (½MIC) as was evidenced by the anti-GFP Western blot (*n* = 4); DMSO-treated *C. albicans* cells were used as the control.

The treatment of *C. albicans* with antifungals and glucose can increase the expression of the ABC transporters [33,34]. Fluorescent microscopy (Figure 5) showed a stronger signal from the green fluorescent protein (GFP)-tagged Cdr1p in the cells that had been incubated with SQLs than in the control without the compounds that were tested. To confirm this observation, we performed Western blot experiments with crude protein extracts that were obtained from cells that were incubated with SQLs (Figure 6). The Cdr1p-GFP protein level in these extracts was also higher in the treated cells than in control.

Among them, **SQL 2** was the most effective inductor of Cdr1p-GFP protein synthesis followed by **SQL 1** and **SQL 4**. On the other hand, **SQL 3** caused reduction in Cdr1p-GFP protein level in the cells.

## 3. Discussion

Based on our experience with SQLs, we decided to use acetylation as the protection of hydroxyl groups. Although hydrolysis of acetoxy group may be rapid, it depends on structure and condition [35]. The same can be found in enzyme-dependent hydrolysis as in lipases present in *Candida* spp. [36]. We found that acetylated styrylquinolines are rather resistant to decomposition in solvent and in the cell. The same was reported also in mammalian cells during in vitro examination of anticancer activity [9,37]. Even during prolonged (96 h) incubation, derivatives with hydroxyl groups are more than ten times active than their acetylated representatives. Moreover, low yield of **SQL 2** (15%) can also be associated with incomplete hydrolysis of **SQL 1** in a pyridine/water environment, and problems with isolation and proper purification of the compound to obtain high purity (multiple crystallization). An alternative method for obtaining styrylquinolines is synthesis in the microwave field, where the reaction environment is excess of aldehyde as in the case of **SQL 4**. The microwave method significantly reduces the reaction time and resolves the disadvantages associated with the need for hydrolysis. Unfortunately, it is generally only effective when the aldehyde is a liquid or has a low melting point as reported elsewhere [31]. Otherwise, this method leads to a mixture of substrates and product (as in the case of **SQL 4**, yield 9%), without solving problems with the isolation and purification of final compounds.

Antifungal activity of styrylquinolines that were synthesized was tested against *C. albicans* strains of different expression of Cdr efflux pumps. The observed activities were only partially in agreement with our other investigations in which the free 8-OH group seems essential for activity. On the other hand, the 4-OH in the styryl part seemed to have an effect that was similar to the 4-OMe and 4-OEt substituents regardless of the quinoline part [10,38]. It is widely documented that the -OH group plays an important role in destroying fungal cells [39,40,41]. Some compounds that contain the phenolic groups can reduce ergosterol biosynthesis and damage the cell membrane of *C. albicans* [42] or bind to chitin in the cell wall [43]. In this regard, the 8-OH/4-OH derivative expressed some interesting patterns of activity that are worth of further investigation. The same level of activity of **SQL** **3** against mutants without Cdr1p and both pumps (Table 1) indicated its role as a substrate of Cdr1p. Unlike other tested compounds, AmB showed an MIC50 at the same concentrations for all strains, suggesting that it is not a substrate for ABC transporters.

In our previous investigations, we observed different influences on the Cdr1 transporter when combinations of FLC-beauvericin and diS-C3(3)-beauvericin were applied in which beauvericin was an inhibitor of the ABC transporters and diS-C3(3) was the substrate for these pumps [44]. Similar differences in inhibitory activity were observed for curcumin [45], the modulatory effect of which was restricted to R6G or miconazole, while it had no effect on the efflux of FLC. Larger flat, aromatic and highly lipophilic compounds, such as styrylquinolines, are known to accumulate in the cell membranes. To confirm that the cell membrane of *C. albicans* was not disrupted by **SQL 3** and **SQL 4**, for which the efflux of rhodamine was higher than the control, the cells that had been treated with styrylquinolines were stained with non-permeable propidium iodide dye [23,46] and observed under a fluorescent microscope. **SQL 1**, **SQL 2, SQL 3**, and **SQL 4** did not show the red staining that is characteristic of membrane disruption (data not shown). Confirmation of this findings was elaborated by means of measuring expression level of Cdr1p-GFP in crude extracts. Surprisingly, it appeared that the **SQL 3** compound reduced the amount of Cdr1p in the cells (Figure 6). Taking into account all of the data that were obtained for this compound renders this observation even more interesting. The **SQL3** showed the best antifungal activity of all tested styrylquinolines both alone (Table 1) and in combination with fluconazole (Table 2). **SQL1**, **SQL2**, and **SQL4** increased both the level of gene expression and the amount of Cdr1 in *C. albicans* cells (Figure 6) which probably contributed to the high resistance of this fungus to these compounds (Table 1).

To sum up, we designed and synthesized four styrylquinolines in order to verify the hypothesis of the importance of the hydroxyl groups in the quinoline and phenyl moieties. All of the compounds were tested against the *C. albicans* wild type and mutants that had been deprived of one or two ABC efflux pumps. According to the expectations, the **SQL 3** compound with both hydroxyl groups that were unprotected appeared to be the most active against the tested strains. Its activity pattern suggested efflux through the Cdr1 pump. All of the compounds that were obtained were also tested in combination therapy with a known Cdr substrate,FLC. The synergistic interactions and activity were strongly reliant on the substitution pattern. Additional experiments will enable us to determine novel inhibitors of the Cdr1p protein.

## 4. Materials and Methods

### 4.1. Compounds

(*E*)-2-[2-(4-acetoxyphenyl)vinyl]-8-acetoxyquinoline (**SQL 1**). Yield 67%, mp. 157–159 °C; ^1^H-NMR (400 MHz, CDCl_3_-*d*) δ 8.15 (dd, *J* = 8.5, 5.1 Hz, 1H), 7.73–7.60 (m, 5H), 7.55–7.37 (m, 3H), 7.19–7.12 (m, 2H), 2.58 (s, 3H), 2.35 (s, 3H); ^13^C-NMR (101 MHz, CDCl_3_-*d*) δ 169.84, 169.33, 155.65, 150.91, 147.38, 140.94, 136.41, 134.25, 133.71, 131.21, 129.10, 128.60, 128.32, 125.75, 125.54, 121.95, 121.68, 120.19, 21.17, 21.03 [47].

(*E*)-2-[2-(4-acetoxyphenyl)vinyl]quinolin-8-ol (**SQL 2**). Yield 15%, mp. 147–149 °C; ^1^H-NMR (400 MHz, DMSO-*d*_6_) δ 9.56 (s, 1H), 8.29 (t, *J* = 10.8 Hz, 1H), 8.14 (d, *J* = 16.2 Hz, 1H), 7.83–7.72 (m, 3H), 7.53–7.42 (m, 1H), 7.43–7.33 (m, 2H), 7.22 (t, J = 5.6 Hz, 2H), 7.15–7.05 (m, 1H), 2.30 (s, 3H); ^13^C-NMR (126 MHz, DMSO-*d*_6_) δ 169.65, 153.79, 153.40, 151.09, 138.61, 137.00, 134.66, 133.83, 128.65, 128.57, 128.17, 127.55, 122.83, 121.46, 118.05, 111.70, 21.36 [9].

(*E*)-2-[2-(4-hydroxyphenyl)vinyl]quinolin-8-ol (**SQL 3**). Yield 63%, mp. 156–157 °C; ^1^H-NMR (400 MHz, DMSO-*d*_6_) δ 11.81 (s, 1H), 10.38 (s, 1H), 8.84 (d, *J* = 8.9 Hz, 1H), 8.42 (d, *J* = 8.9 Hz, 1H), 8.22 (d, *J* = 16.2 Hz, 1H), 7.79 (d, *J* = 16.3 Hz, 1H), 7.67–7.56 (m, 4H), 7.47 (dd, *J* = 6.1, 2.6 Hz, 1H), 6.95 (d, *J* = 8.6 Hz, 2H); ^13^C-NMR (126 MHz, DMSO-*d*_6_) δ 161.19, 153.30, 148.85, 144.14, 143.99, 143.72, 130.85, 129.57, 128.20, 126.55, 119.16, 118.74, 116.83, 116.50 [47].

(*E*)-2-[2-(4-hydroxyphenyl)vinyl]-8-acetoxyquinoline (**SQL 4**).

Step 1. 8-Acetoxyquinaldine: The 8-hydroxyquinaldine derivative (2.5 mmol) in 10 mL acetic anhydride was heated for 16 h at 130 °C. Then, the mixture was evaporated to dryness and a solid was crystallized from EtOH to produce a light yellow solid, yield 95%, mp. 67 °C; ^1^H-NMR (400 MHz, CD_2_Cl_2_-*d*_2_) δ 8.12 (d, *J* = 8.5 Hz, 1H), 7.74 (dd, *J* = 8.2, 1.3 Hz, 1H), 7.50 (t, *J* = 7.8 Hz, 1H), 7.42 (dd, *J* = 7.5, 1.3 Hz, 1H), 7.37 (d, *J* = 8.5 Hz, 1H), 2.74 (s, 3H), 2.48 (s, 3H).

Step 2. (*E*)-2-[2-(4-hydroxyphenyl)vinyl]-8-acetoxyquinoline: The appropriate quinaldine derivative (1 mmol) was mixed thoroughly with two equivalent aldehyde, put into an open vessel, and exposed to microwave irradiation for 20 min at 180 °C with a maximum power 80 W. Then, the reaction mixture was cooled to 0 °C and the precipitate was filtered off. The solid was recrystallized from EtOH in order to produce a dark yellow solid, yield 9%, mp. 132–136 °C; ^1^H-NMR (500 MHz, DMSO-*d*_6_) δ 9.98 (s, 1H), 8.27 (t, *J* = 11.4 Hz, 1H), 8.10 (d, *J* = 16.4 Hz, 1H), 7.79 (d, *J* = 8.6 Hz, 1H), 7.66–7.59 (m, 1H), 7.56–7.48 (m, 1H), 7.40–7.32 (m, 2H), 7.21–7.13 (m, 1H), 7.13–7.04 (m, 1H), 6.95 (t, *J* = 8.8 Hz, 1H), 6.88 (t, *J* = 7.4 Hz, 1H), 1.91 (s, 3H); ^13^C-NMR (126 MHz, DMSO-*d*_6_) δ 169.69, 153.80, 153.38, 151.08, 138.60, 137.01, 134.64, 133.82, 128.66, 128.59, 128.17, 127.56, 122.82, 121.42, 118.07, 116.31, 111.71, 21.35.

### 4.2. Strains and Growth Media

The *C. albicans* strains used in this study were a generous gift from Dominique Sanglard (Table 3). All of the strains were kept as frozen stocks in glycerol at −80 °C and routinely grown at 28 °C on a YPD medium with 2% dextrose, 1% Bacto peptone (Diag-med, Warsaw, Poland) and 1% yeast extract (Diag-med, Warsaw, Poland), and a Sabouraud medium (4% dextrose, 1% Bacto peptone (Diag-med, Warsaw, Poland). For all of the experiments, except for a Rhodamine 6G (Sigma–Aldrich, Poznan, Poland) assay, the strains were sub-cultured in w yeast nitrogen base (YNB) liquid medium (0.67% YNB (Diag-med, Warsaw, Poland) and 2% dextrose) at 28 °C and diluted to the desired optical density at 600 nm (OD600). For growth on solid media, 2% agar (Difco) was added.

### 4.3. Susceptibility Testing

Drug susceptibility testing was performed in microtiter plates with two-fold serial dilutions of the tested compounds according to the EUCAST (EUCAST E.DEF 7.3) specifications with modifications. The cultures were grown for 18 h on Sabouraud agar at 35 °C, and a few colonies were suspended in 0.85% NaCl at 0.5 (1.5 × 10^6^ CFU/mL) in the McFarland scale. Next, the suspension was diluted to a final concentration of 2.5 × 10^5^ CFU/mL and 100 µL was mixed with 100 µL of the tested compound suspended in 2 × YNB or a mixture of FLC (Sigma–Aldrich; Poznan, Poland) and styrylquinoline, each at a starting concentration of MIC in order to obtain the fractional inhibitory concentration (FIC) indexes. Those indexes are determining type of interaction between two drugs, where: synergy: FICI ≤ 0.5, indifference: FICI > 0.5 to 4, antagonism: FICI > 4 [51]. The FIC index was defined as the MIC of drug “a” applied in combination with drug “b”, divided by the MIC of drug “a”, added together with the MIC of drug “b” applied in combination with drug “a”, divided by MIC of drug “b” according to the formula below (Equation (1)):

FICI = MIC_a+b_/MIC_a_ + MIC_b+a_/MIC_b_ (1)

Microtiter plates were incubated at 28 °C for 24 h and then shaken, and their optical densities were read with a microtiter plate reader at a wavelength of 600 nm. The results are presented as the percentage of growth relative to the control samples, and the MIC values are given as the lowest concentration that inhibited 50% of the growth according to the EUCAST standards. The experiment was performed in three biological replicates with three technical replicates.

### 4.4. Permeabilization Assays

The yeast cultures were sub-cultured overnight in YNB and diluted to an OD600 of 0.4 in a fresh medium. The styrylquinoline compounds were added and incubated at 28 °C with shaking at 180 rpm. Aliquots of the cultures were taken after 4 h and stained with propidium iodide (manufacturer: Bioshop, distributor: Lab Empire, Rzeszów, Poland) [23,46] for 5 min at room temperature in order to assess the membrane permeability. Observations were made using fluorescent microscopy (Zeiss AxioVision, Poznań, Poland). The experiment was carried out in three biological replicates.

### 4.5. Western Blotting

The assay was performed according to a previous method [52] with modifications. A crude protein extract was prepared from the cell suspensions after 4 h of induction with the tested compounds. The aliquots of the cell suspensions were pelleted via centrifugation at 2260× *g* for 5 min and resuspended in 1 mL of deionized water. The cells were lysed by adding 150 μL of 1.85 M NaOH-7.5% β-mercaptoethanol (Sigma–Aldrich, Poznan, Poland) and incubated on ice for 10 min. The proteins were precipitated by adding 150 μL of 50% trichloroacetic acid and incubated on ice for 10 min. Samples were then centrifuged at 10,000× *g* for 5 min at 4 °C, washed in 1 mL of 1 M Tris-HCl pH 8.0, and then resuspended in 50 μL of the sample buffer (40 mM Tris-HCl, 8 M urea, 5% SDS, 0.1 mM EDTA 1% β-mercaptoethanol, 0.1 mg/mL bromophenol blue). Following incubation at 37 °C for 30 min, the samples were loaded onto 6% sodium dodecyl sulphate-polyacrylamide gel and developed in a Mini-PROTEAN II electrophoresis cell (Bio-Rad, Poznań, Poland). The samples were then transferred onto a nitrocellulose membrane using a Mini-PROTEAN Tetra System electrophoresis cell (Bio-Rad, Poznań, Poland). The membranes were stained with Poncau S to check whether the gels had loaded equally. The Cdr1p was immunodetected using polyclonal mouse anti-GFP antiserum with horseradish peroxidase-conjugated anti-mouse antiserum as a secondary antibody. The signals were detected using an ECL kit from PerkinElmer according to the manufacturer’s instructions. The experiment was performed in four biological replicates.

### 4.6. Rhodamine 6G Assay

The R6G assay was performed according to Nakamura et al. [53] with modifications. The cell culture was pelleted in the log phase and washed twice in double-distilled water and once in a HEPES buffer (50 mM, pH 7.0). The cells were diluted in a fresh HEPES buffer to an OD600 of 1.0 and incubated for 60 min at 30 °C and 200 rpm with 5 mM 2-deoxy-d-glucose (Sigma–Aldrich, Poznań, Poland). Next, the tested compounds were added in ½MIC concentrations and the cells were incubated for 5 min in the same conditions. Then, 10 µM R6G was added and the cell suspension was incubated for an additional 90 min. The cells were pelleted, washed twice in a HEPES buffer, and suspended in fresh HEPES at an OD600 of 10.0. The cell suspension was incubated for 5 min at 30 °C with shaking, the R6G efflux was initiated by adding 10 mM glucose and the suspensions were incubated for 30 min from which aliquots were removed at 15 min intervals. Aliquots of 400 µL were pelleted and three duplicates of 100 µL of supernatant were added to black microtiter plates. The fluorescence was measured in a Cary Eclipse spectrofluorimeter (Agilent Technologies, Santa Clara, CA, USA) at an excitation wavelength of 529 nm (slit 5) and an emission of 553 nm (slit 10). The experiment was performed in three biological replicates with three technical replicates.

## Figures and Tables

**Table 1:** The minimum inhibitory concentration (mg/L) at which 50% of isolates were inhibited (MIC50) for tested styrylquinoline compounds and amphotericin B (AmB) (*n* = 3).

Compound	WT (Wild Type Strain)	*cdr1Δ*	*cdr2Δ*	*cdr1Δcdr2Δ*
**SQL** **1**	>138.8	>138.8	>138.8	138.8
**SQL** **2**	>122.12	>122.12	>122.12	122.12
**SQL 3**	26.33	3.29	26.33	3.29
**SQL 4**	>122.12	>122.12	>122.12	122.12
**8HQ**	17.42	36.29	17.42	17.42
**8HQD**	19.10	39.79	19.10	19.10
**AmB**	0.9241	0.9241	0.9241	0.9241
**Fluconazole**	18.37	0.490	0.980	0.244

**Table 2:** The fractional inhibitory concentration (FIC) values for styrylquinoline compounds (*n* = 3) that were tested.

Compound	WT (Wild Type Strain)	*cdr1Δ*	*cdr2Δ*	*cdr1Δcdr2Δ*
**SQL 1**	2	1	2	1
**SQL 2**	2	0.5	2	0.5
**SQL 3**	0.02	0.02	0.02	0.02
**SQL 4**	1	0.5	0.5	0.5

**Table 3:** Collection of *C. albicans* strains that was used in this study. Strain CAF 2-1 is treated as a wild-type (WT) strain. The deleted genes encoding drug efflux pumps (Cdr1, Cdr2 and Mdr1) are marked delta in the Genotype column.

Strain	Genotype	Reference
CAF 2-1	*ura3Δ::imm434/URA3*	[48]
DSY 448	*cdr1Δ::hisG-URA3-hisG/cdr1Δ::hisG*	[49]
DSY 653	*cdr2Δ::hisG-URA3-hisG/cdr2Δ::hisG*	[50]
DSY 654	*cdr1Δ::hisG/cdr1Δ::hisG cdr2Δ::hisG-URA3-hisG/cdr2Δ::hisG*	[50]
